# Physician Perspectives on Reducing Curative Cancer Treatment Intensity for Populations Underrepresented in Clinical Trials

**DOI:** 10.1093/oncolo/oyac191

**Published:** 2022-10-10

**Authors:** Gabrielle B Rocque, Courtney Andrews, Valerie M Lawhon, Stacey A Ingram, Rachel M Frazier, Mary Lou Smith, Lynne I Wagner, Lisa Zubkoff, Lauren P Wallner, Antonio C Wolff

**Affiliations:** Division of Hematology and Oncology, Department of Medicine, University of Alabama at Birmingham, Birmingham, AL, USA; Division of Gerontology, Geriatrics, and Palliative Care, Department of Medicine, University of Alabama at Birmingham, Birmingham, AL, USA; O’Neal Comprehensive Cancer Center, Birmingham, AL, USA; Institute for Human Rights, University of Alabama at Birmingham, Birmingham, AL, USA; Division of Hematology and Oncology, Department of Medicine, University of Alabama at Birmingham, Birmingham, AL, USA; Division of Hematology and Oncology, Department of Medicine, University of Alabama at Birmingham, Birmingham, AL, USA; Division of Hematology and Oncology, Department of Medicine, University of Alabama at Birmingham, Birmingham, AL, USA; Research Advocacy Network, Plano, TX, USA; Wake Forest School of Medicine, Winston Salem, NC, USA; O’Neal Comprehensive Cancer Center, Birmingham, AL, USA; Division of Preventive Medicine, , Department of Medicine, University of Alabama at Birmingham, Birmingham, AL, USA; Birmingham/Atlanta Geriatric Research Education and Clinical Center (GRECC), Birmingham VA Healthcare System, Birmingham, AL, USA; Rogel Cancer Center, Departments of Internal Medicine and Epidemiology, University of Michigan, Ann Arbor, MI, USA; The Johns Hopkins University School of Medicine, Baltimore, MD, USA

**Keywords:** physician perspectives, clinical trials, de-escalation, de-implementation, breast cancer, physician perspectives, qualitative

## Abstract

**Background:**

Historically, clinical trials involved adding novel agents to standard of care to improve survival. There has been a shift to an individualized approach with testing less intense treatment, particularly in breast cancer where risk of recurrence is low. Little is known about physician perspectives on delivering less intense treatment for patients who are not well represented in clinical trials.

**Methods:**

Open-ended, individual qualitative interviews with medical oncologists explored their perspectives on trials that test less intense treatment for patients with cancer, with a focus on breast cancer. Interviews were audio-recorded and transcribed. Four independent coders utilized a content analysis approach to analyze transcripts using NVivo. Major themes and exemplary quotes were extracted.

**Results:**

Of the 39 participating physicians, 61.5% felt comfortable extrapolating, 30.8% were hesitant, and 7.7% would not feel comfortable extrapolating trial outcomes to underrepresented populations. Facilitators of comfort included the sentiment that “biology is biology” (such that the cancer characteristics were what mattered), the strength of the evidence, inclusion of subset analysis on underrepresented populations, and prior experience making decisions with limited data. Barriers to extrapolation included potential harm over the patient’s lifetime, concerns about groups that had minimal participants, application to younger patients, and extending findings to diverse populations. Universally, broader inclusion in trials testing lowering chemotherapy was desired.

**Conclusions:**

The majority (92%) of physicians reported that they would de-implement treatment for patients poorly represented in clinical trials testing less treatment, while expressing concerns about applicability to specific subpopulations. Further work is needed to increase clinical trial representation of diverse populations to safely and effectively optimize treatment for patients with cancer.

**Trial registration:**

NCT03248258

## Teaser

This article explores physicians’ perspectives on extrapolating results to patients underrepresented in cancer clinical trials that test less intense treatment for patients, focusing on breast cancer.

**Implications for Practice**

Historically, clinical trials involved adding novel agents to standard of care to improve survival. There has been a shift to an individualized approach testing less intense treatment. The majority (92%) of oncologists reported that they would de-implement treatment for patients poorly represented in clinical trials testing less treatment, while expressing concerns about applicability to specific subpopulations. Further work is needed to increase clinical trial representation of diverse populations to safely and effectively optimize treatment for patients with cancer.

## Introduction

Clinical trials provide the foundation for evidence-based practices, yet participants within these trials are often not representative of the general population. Unger and colleagues estimated that fewer than 1 in 20 patients in the US participate in clinical trials, and those participants are typically medically fit, younger, and healthier than the general population.^1^ Furthermore, in prior analyses of patients with early stage breast cancer, as many as half of patients receiving care in real-world settings would be considered underrepresented due to age, race, ethnicity, and/or comorbidities.^2,3^ Lack of representation is problematic, as prior studies demonstrated differences in treatment intensity^2^ and, in some cases, survival differences for patients who were well represented vs. those who have traditionally been poorly represented.^3^ Thus, learning how evidence generated from clinical trials is applied by physicians to broader populations with greater diversity is paramount.

The historical paradigm of clinical trials adding novel agents to improve survival is shifting toward a more individualized approach to treatment based on patients’ risk profiles, with the emergence of clinical trials testing less intense treatment approaches.^4^ This approach is particularly prominent in early stage breast cancer,^5^ where risk of recurrence is low and long-term toxicity is burdensome. They have the potential to spare toxicity, with the potential risk of reduced efficacy, potentially leading to recurrence and death. This approach is notably different from trials testing more intense treatment where the potential benefit is improved survival at the risk of greater toxicity. This new approach may exacerbate known disparities in clinical trial enrollment. In prior qualitative work, patients cited concerns about receipt of less than standard of care.^6^ This was particularly true for Black women, given the historical atrocities experienced by the Black community, including the Tuskegee experiments depriving patients with syphilis of standard of care.^7^ One participant highlighted that “Tuskegee has a major impact on African American women” when considering participating in research testing less treatment.^6^ In addition, several patients reported that de-implementation clinical trials would be less attractive for younger, healthier patients who would be willing to tolerate additional toxicity for a potential cure.^6^ Thus, traditional framing of older adults with comorbidities not being adequately represented may be reversed. There is emerging data to suggest that enrollment for de-implementation trials differs from trials adding treatments, with trials testing less intense therapy having greater numbers of older adults and fewer patients with more advanced stages of disease. When the adjuvant paclitaxel and trastuzumab (APT) phase II trial single arm tested a less intense treatment approach in early-stage breast cancer than is typically administered, 10% of patients were 70 years or older. In addition, while patients with both T1 (<2 cm) and T2 (2-5 cm) tumors were eligible, only 9% of patients had T2 tumors within the sample.^8^ Thus, physicians must consider differences between clinical trial participants and the patients they care for in routine practice.

If optimization trials have favorable results, the excess treatments must be de-implemented in the real world. De-implementation is defined as “reducing or stopping the use of a health service or practice provided to patients by health care practitioners and health care delivery systems”.^9^ In the absence of data for underrepresented patient groups from trials, physicians must decide whether to de-implement and how to discuss applicability of existing trial data with patients. Given the potential for inferior efficacy, the scientific rigor for application into real-world populations should be high. Little is known about physician perspectives on de-implementing less intense treatment approaches for patients who are not represented in sufficient number to draw conclusions on subpopulations. The objective of this analysis was to explore physician perspectives on de-implementing treatment for patients who were not well represented within the trials testing less intense treatment.

## Materials and Methods

The Consolidated Criteria for Reporting Qualitative Studies (COREQ) was leveraged for reporting of results.^10^

### Research Team and Reflexivity

The study design was developed and led by a breast medical oncologist with expertise in qualitative research (GR). The research team included a medical anthropologist (CA), a medical student (RF), researchers with a public health degrees (VL, SI), an epidemiologist (LW), a psychologist (LW), a patient advocate (MS), an implementation science expert (LZ), and 2 oncologists (GR, AW). The interviews were conducted by a medical oncologist (GR).

### Study Design and Recruitment

This study is part of a larger qualitative study exploring academic and community medical oncologist perspectives on studies that test less intense treatment for cancer patients, with an emphasis on breast cancer. This study reported here focuses on questions surrounding applicability of results to real-world populations that were not well represented within clinical trials. A convenience sample of participants from unique practices was identified through membership in cooperative groups, prior working relationships with the oncologists, or referral from prior participants. Purposive sampling was leveraged to balance work setting (academic vs. community) and sex. Emphasis was placed on identifying participants with diverse time practicing medical oncology, geographic location, and racial and ethnic background. Inclusion criteria consisted of medical oncologists age ≥ 18. For academic oncologists, all participants had a breast cancer focus. The PI invited eligible physicians via email to participate in a brief (30-60 minute) interview. Informed consent was implied by interview participation. No monetary compensation was provided. A sample size of up to 40 physicians was planned, and enrollment was continued until thematic saturation was reached.^11,12^ The University of Alabama at Birmingham Institutional Review Board approved this evaluation.

### Data Collection

Participants completed a short demographic questionnaire, including basic demographics. Additional information about practice locations were abstracted from webpages about their institution. Physician interviews were conducted over Zoom or telephone and recorded. A semi-structured interview guide focused on perspectives on reducing treatment intensity for patients (Supplemental Appendix A). Guides were developed using an a priori model based on Norton and colleague’s De-implementation Framework^9^ and aligned with patient interview guides from a prior published project.^6^ Interviews were professionally transcribed and verified by the research team.

### Analysis

Four independent coders (C.A., V.L., R.F., G.R.) reviewed transcripts for this analysis and coded using QSR International’s NVivo 11.4.3. Along with the principal investigator (PI) (G.R.), two coders (C.A., V.L.) generated an analytic codebook based on a priori and emergent codes. Codes were reviewed every 2-4 transcripts using a constant comparative method to determine when coding saturation was reached and to ensure coding consistency. For the initial 10 transcripts, they were also reviewed with the PI (G.R.) to review clinical context. The final codebook was reviewed and agreed upon by all coders and the study PI. Discrepancies were reviewed and discussed until consensus was reached. Key themes and exemplary quotes were identified and investigator insights relevant to the decision-making process were noted in memo format.

## Results

Of the 46 physicians approached, 39 participated in the study between March 31, 2021 and November 5, 2021. Seven did not respond to email request or declined due to lack of time. The sample was drawn from 17 states, each from a different oncology practice. Physicians were balanced for men vs. women (49% vs. 51%), community oncologist vs. academic oncologist (49% vs. 51%), and time practicing as medical oncologist (31% 0-9 years; 33% 10-19 years; 36% 20+ years). Amongst participants, 8% of physicians were Black, 8% Hispanic, 10% Asian, and 74% White. Complete demographics are shown in **Table 1**.

**Table 1.** Oncologist demographics.

Characteristic	*n* (%) or median (range)
Sex	
Male	19 (51%)
Female	20 (49%)
Race and ethnicity	
White/Non-Hispanic	29 (74%)
Asian	4 (10%)
Black	3 (8%)
White/Hispanic	3 (8%)
Age, years, median (range)^a^	50 (34-78)
Practice type	
Academic	20 (49%)
Community^b^	19 (49%)
Years as an oncologist^c^	
<5	4 (10%)
5-10	8 (21%)
10-19	13 (33%)
20-29	6 (15%)
30+	8 (21%)

Age = 2021—birth year.

Community practice includes community practices with academic affiliation.

Years as oncologist = 2021—year started practice.

### Physicians Perspectives on Why They Would Be Comfortable Extrapolating Data

A substantial proportion (24; 61.5%) of physicians were comfortable extrapolating clinical trial results to patients not well represented based on patient characteristics (eg, age or race), despite a general sentiment that lack of representation was not ideal. An additional 12 (30.8%) were comfortable extrapolating in some, but not all cases. At times, physicians expressed frustration with pressures to adhere only to the clinical trial population, with one physician stating, “we should stop punishing each other about all these deviations that don’t impact anybody, but limit how many people we can help at the end of it (Physician 17)”.

**Figure 1.**
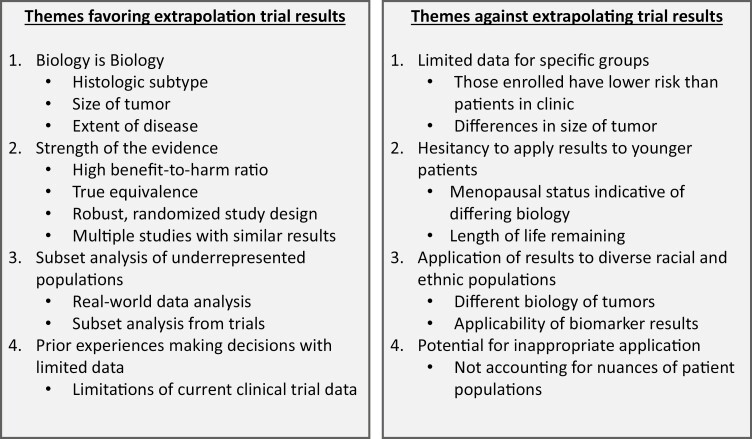
Themes favoring and against extrapolation of clinical trial results to de-implement treatment for underrepresented populations.

Four key themes emerged for why physicians would apply clinical trial results more broadly (**Figure 1**). The first was the concept “*the biology is the biology* (Physician 1).” Providers emphasized the tumor histological subtype (triple negative, estrogen-receptor positive, or Her2-neu positive) and tumor size as the main drivers of biology. For many physicians, the concept of biology superseded issues surrounding age or race. This sentiment was shared by multiple physicians: “assuming that we have captured a fair distribution of the biology that we’re trying to deescalate, I think that’s okay . . . I don’t know if I would be worried if it’s about age, as long as we capture the biology (Physician 6)” and “as long as biologically the tumors look the same, I would apply at face value to patients of different age groups and race. Because it’s all about the biology of the cancer (Physician 16).” She further went on to explain how she would apply data from older women to support decisions to de-escalate treatment for younger women with comorbidities to avoid toxicity: “‘There weren’t lots of people like you in this study. But I feel like because of this study, the risks outweigh the benefits of including that drug for you and that we can safely do this.’ If anything, it gives you more flexibility to deescalate in those other populations (Physician 16).”

A second key theme was the strength of the evidence generated from the trials. One physician emphasized the importance of the science in his willingness to apply results to a general population, “the scientific rigor of the work, especially since you’re going to be making a somewhat seismic shift in oncologists’ natural inclination to aggregate, to pile on (Physician 13).” The strength of the evidence included considerations of both harms and benefits, “I’m comfortable extrapolating down depending how beneficial was the outcome compared to the standard treatment (Physician 8).” Physicians hoped to see not only statistical equivalence, but true equivalence with equal survival percentages in a non-inferiority trial. “it also has to depend on how the data looks . . . If the percentages were exactly the same on a non-inferiority trial, I feel a little bit better about it (Physician 16).” In addition, the design also contributed to believability of results. Multiple participants discussed the importance of randomization to reduce bias, “You want to be sure that there will not be a selection bias that creates asymmetric populations that are not truly apples for apples. That would be very important. So I do think that randomization would be a requirement (Physician 13).” Another physician talked about a specific study that did not have a control group, “We don’t have a control group so that we’re not going to really know how these patients would have done if they were treated differently; so I think it’s going to be a challenge (Physician 16).” A third physician noted, “There is still the need to follow the scientific method. For me, a trial is really frustrating when I can’t interpret it correctly, because there are so many ifs, ands, and buts about it that make it hard to trust. A trial that only creates a need to do another trial to answer the same question is really about the most damnable form of clinical trial research there is.” Additionally, the cumulative evidence of safety across multiple studies and populations was important, “It really depends upon the overall depth and breadth of the data. If you have one clinical trial where you have a small population that is enrolled, but it seems to benefit. And you have another one in another clinical trial with that same small group, but they also seem to benefit. You’re going to feel more confident (Physician 32).” One physician recommended a stepped approach to expanding the populations, “You may have to have a step design, where you do it in a less risky population, and then say, ‘Okay. We proved our concept, now we’re going to look at it in a broader population’.”

Third, subset analysis on underrepresented subpopulations could facilitate confidence with broad use of de-escalation. For example, one physician shared the importance of real-world data, stating, “Real-world data can sometimes be useful to help address those knowledge gaps. As a general rule, though, if there’s been a therapeutic advance that’s been identified in a population, even if that population does not exactly represent the makeup of the patient in front of me, we’ll still inform the patient that this is the standard of care, it’s been informed by this trial, in this population (Participant 29).” Another physician commented on the importance of subset analyses and propensity scoring to enhance the perception of generalizability.

Finally, physicians acknowledged their prior experience making decisions with limited data, with physicians noting, “realistically, no trial will ever be representative of the population (Physician 8)” and “Well, all of it’s extrapolation. The average age of people on clinical trials is 10 years younger than those who were not on clinical trials. And so, I mean, as much as possible you want to match up the indications, basically the parameters of the study against the patient that you have . . . you got to use what you got (Physician 18).” While many participants acknowledge decision-making without adequate data, some participants lamented this reality, stating, “We’ve been extrapolating for as long as I can remember and certainly that I’ve been in practice. So we do need to do better there, but extrapolation is only natural with what we have, the limitations we have (Participant 33)” and “practically speaking in day to day clinic, you don’t have much of a choice. It’s what we have. It’s insufficient. And, it’s really unfortunate (Physician 24).”

### Physician Perspectives on Why They Would Be Uncomfortable Extrapolating Data

Fifteen (38.7%) providers expressed hesitancy, of varying degrees, to extrapolate data to populations not adequately represented in clinical trials; twelve stating this was dependent on the situation whereas three reported they would not extrapolate at all. They noted several key themes regarding concerns applying these clinical trials (**Figure 1**). A few physicians dogmatically applied clinical trial results, with one explaining, “as a physician looking at a study . . . it would be difficult for me if I felt like the demographic was not consistent with my demographic that I wanted to apply it to (Physician 37).” Others expressed hesitancy, while acknowledging that they apply results for some patients, “We are going to have a hard time extrapolating some of that data to those that are less represented (Physician 28).” For some, these reservations were blanketed for *any group who had limited data*, “the groups of patients [I am] most reluctant to do it would be the groups where we have the fewest data (Physician 32).”

Other physicians focused on specific patient characteristics that were concerning. The most common concern voiced was hesitancy to apply results to younger patients. When asked who she would be most uncomfortable applying results to, one participant said, “very, very young women (Physician 33).” Physicians felt that more patient data was needed: “In general, most clinicians tend to be a bit more aggressive with the younger patients (Physician 32).” The concerns about applying to younger populations were linked to other factors. For example, some providers considered age to be more important for women with ER+ breast cancer due to the association of younger age with menopausal status. One physician commented, “if you did a de-escalation study and most of the people were post-menopausal and then you want to bring it into the premenopausal setting, I’d have to give it thought (Physician 8).”

The potential to harm patients over the lifetime was another key theme, particularly for younger women: “if we screw up, is this [going] to affect your quality of life, your quality of life in a substantial way? And if the answer is yes, then I’m like, hmm, a little bit more nervous in extrapolating something that was really underrepresented (Physician 4).” This provider went on to consider the importance of age in the risk for harm, stating, “Skimping out may actually not have an impact on survival for somebody who’s already in their mid-seventies, eighties, or whatnot. But for somebody in their thirties, they recur when they’re 60, that still sucks (Physician 4).” Another physician echoed this concern, “There’s a big difference for a woman who has a 10-year life expectancy, versus a woman who has a 40-, 50-year life expectancy, which is a big difference in how you think through treatment decisions (Physician 12).”

In addition to age, some physicians expressed worry about application of results to diverse racial and ethnic populations: “Nationwide, we don’t have enough Black patients participating in trials. And so sometimes it’s like, ‘Can we extrapolate this data or not? We don’t know.’ It’s an understudied population (Physician 1).” Another physician commented, “I’m very uncomfortable with that, and so I think that there has to be targeted enrollment to make sure that there is adequate representation in these trials. Or we just have to be really clear about in whom we can apply the results (Physician 10).” Physicians expressed concerns that patients of different racial and ethnic backgrounds might have differing biology contributing to disparities, “We know these tumors biologically for certain ethnicities are different (Physician 33)” and **“**Asians and African-Americans definitely [have] worse prognosis. And yes, although it is talked about, socioeconomic reasons as a reason, but there is some maybe harder biology there too that we don’t quite understand (Physician 11).”

### A Call for Broader Representation in Clinical Trials

Physicians in this study routinely highlighted a need to expand clinical trial enrollment to more diverse populations, “the hope is that we can enroll a very diverse population. And I think that should be a focus . . . a concerted effort should be made, whether it’s having enrollment for certain populations to diversity of the patient population (Physician 9)” and “it just underscores why there should be a great effort to try to somehow recruit those patients into these trials. It’s hard to do, you know that. But, at the grassroots level, I think that’s going to be really, really important (Physician 28).” Physicians noted that this was a priority for not only the individuals, but also the cooperative groups, “We have to go to eliminate these issues in a clinical trial design. The mindset to accomplish that and the drive and the willingness to be more conscientious of that fact and proactive in extending clinical trials to larger patient populations are certainly very much alive in the cooperative groups (Physician 21).”

## Discussion

To our knowledge, this is the first study that describes physician perspectives on how they consider de-implementation for patients not represented in clinical trials testing less intense treatment for cancer. Importantly, physicians both call for greater representation and provide key actionable insights for clinical trial design to enhance future adoption into practice. Physicians emphasized that robust and rigorous trial design is needed when testing a novel therapeutic approach, particularly one where the potential risk is recurrence and death. The randomized control trial remains the gold standard due to the limitation of unanticipated confounders that may result from a poorly balanced group.^13^ Second, the emphasis on biology and cancer stage, defined by the size of the tumor and the extent of spread, suggests an opportunity for block randomization by stage with capitation of enrollment of smaller tumors to ensure adequate data for more advanced stages. This approach has been successfully used in escalation studies to enrich for high risk patients who will benefit from additional therapy, such as the PALLAS study which capped the enrollment of patients with stage IIA disease in order to select for higher risk patients who would benefit from the addition of palbociclib to adjuvant endocrine therapy for early stage breast cancer.^14^ Other cooperative group studies have exclusively enrolled Black women^15^ or capped enrollment for older adults and White patients to ensure adequate number of younger and Black patients.^16^ In studies testing less treatment, such a limitation would help to define the upper limit of risk at which reducing treatment is safe. This approach also can be used to balance other patient characteristics associated with risk or hesitancy to participate in de-escalation clinical trials (eg, age, race) in order to ensure that results are applicable across the range of eligible patients.

In addition to strategic study design, our study highlights a new population at risk for underrepresentation in clinical trials targeting overtreatment—young women. Although younger women may have higher recurrence risk, overtreatment in this population is particularly problematic due to the potential to live years with physical side effects from chemotherapy, such as chronic neuropathy, heart failure, and risk of secondary cancers.^17^ In addition, young women have other unique toxicity considerations, including fertility, receipt of treatment during pregnancy, and bone loss due to early menopause.^18^ Furthermore, chemotherapy can contribute to lifelong concerns related to sexuality, cognitive changes, and emotional distress.^17^ Thus, optimizing our treatment approach to balance both the risk of long-term durable toxicities that adversely impact quality of life with risk of recurrence is critical for young women.

Another group for whom representation continues to be a challenge are patients of diverse racial and ethnic backgrounds. De-implementation studies may be more prone to low enrollment of Black patients than studies intensifying treatment due to both patient historical mistrust associated with undertreatment on clinical trials^6^ and the physician perceptions noted in this study that Black women have more aggressive disease. Since the National Institute of Health Revitalization Act of 1993 required federally funded research to include minorities in clinical trials, inclusion has become an important strategy in reducing health disparities.^19^ While increased representation of Black women in clinical trials is recommended by the American Society of Clinical Oncology and the Food and Drug Administration,^20,21^ representation is of the utmost importance when trials represent a true paradigm shift in the treatment of cancer. Participation of Black women in de-escalation trials is particularly important because Black women may have greater toxicity from chemotherapy,^22,23^ and are uniquely positioned to benefit. If trials show equivalence of chemotherapy omission in a predominantly older, White population, safety data will be lacking for Black women, particularly in triple negative breast cancer, which disproportionately impacts young, Black women.^24^ In this study, the reluctance of physician to de-implement treatment for underrepresented racial groups, particularly those with higher risk biology, along with the need for additional data on these populations, must be addressed to ensure delivery of optimal treatment that does not overtreat or undertreat patients.

This study brings forth ethical concerns related to application of results of clinical trials without representation. Underrepresentation of ethnically diverse populations in cancer clinical trials may result in the inequitable distribution of the risks and benefits of new forms of treatment.^25^ Despite the NIH inclusion mandate, trial recruitment processes continue to be shaped by socio-structural inequities in the U.S. healthcare system and research infrastructures that create differential access to trials.^26^ Equitable enrollment of historically underrepresented groups in clinical trials requires a collaborative and collective effort that extends beyond the medical institution and involves strategic community engagement and equitable allocation of resources to underfunded research sites.^27^ Beyond physician bias and shared decision making at the individual level, addressing disparities in research participation requires intentional efforts by providers, institutions, and communities to overcome barriers of awareness, opportunities, and acceptance to participate in clinical trials.^28^

This study has potential limitations. Our convenience sampling may have limited the number of participants who would be comfortable with the concept of de-implementation clinical trials. In addition, while we did enroll similar numbers of academic and community oncologists, the study sample focuses on physicians who are engaged in clinical trials. Despite the diverse sample from 39 different clinical practices, this may inadequately represent physicians from smaller, rural practices or who don’t enroll on clinical trials and who may have other considerations regarding application of trial data. Inclusion of a larger sample with greater number of oncologists with more varied backgrounds could provide additional insights. Another limitation is that the examples provided in the interview guide were breast cancer examples, where de-escalation is a substantial focus at present. However, the community oncologists drew from their experience with non-breast cancer examples as well (eg, shortening chemotherapy in colon cancer). Results may be less applicable to other cancer types, particularly where survival rates are lower. Finally, this study did not measure levels of implicit bias related to specific groups (eg, younger patients, Black women) that may be inadequately represented.

## Conclusions

The majority (92%) of physicians report that they would extrapolate clinical trial results to de-implement cancer treatment for patients that are poorly represented in trials in at least some cases, while expressing concerns about applicability to specific subpopulations based on tumor characteristics (eg, stage, biology) and patient demographics (eg, age, race). Increasing clinical trial representation of diverse populations to be able to safely and effectively optimize treatment for patients with cancer is critically needed.

## Supplementary Material

oyac191_suppl_Supplementary_Appendix

## Data Availability

The data underlying this article will be shared on reasonable request to the corresponding author.

## Abbreviations

APT: adjuvant paclitaxel and trastuzumab
COREQ: Consolidated Criteria for Reporting Qualitative Studies
PI: principal investigator
ER+: estrogen receptor positive
HER2: human epidermal growth factor receptor 2
NCCN: National Comprehensive Cancer Network
CR: complete response
THP: tetrahydropyranyladriamycin
