# Supplementary material for: Physician Perspectives on Reducing Curative Cancer Treatment Intensity for Populations Underrepresented in Clinical Trials
Source: Oncologist. 2022 Oct 10;27(12):1067–73. doi: 10.1093/oncolo/oyac191 (PMC9732232; doi:10.1093/oncolo/oyac191)
Supplement: oyac191_suppl_Supplementary_Appendix [file oyac191_suppl_supplementary_appendix.docx]

**Physician Interview Guide Phase III:**

Good [morning/afternoon/evening.]. My name is [TBN] and I will be conducting today’s interview. You have been invited to participate in this study because you provide care for patients with metastatic breast cancer. We are working to better understand what affects decisions for cancer treatment.

We are currently planning clinical trials that test the ability to decrease the amount treatment given to patients. This may result in fewer short-term or permanent side effects from chemotherapy.

For participants at sites with COMPASS open: An example of this is the COMPASS study, which your site is participating in.

For other participants: For example, one study evaluates patients receive neoadjuvant chemotherapy with a single chemotherapy and two HER-targeted therapies instead of two chemotherapies and two Her-2 targeted therapies. For patients with no residual disease at surgery, they do not get more chemotherapy. They can get more chemotherapy if they do not have complete response.

Another example would be to forgo chemotherapy based on a biomarker, such as tumor infiltrating lymphocytes.

We will evaluate if these patients have recurrence rates that are similar to rates reported in prior studies reporting on standard of care regimens.

For today’s discussion, we are interested in hearing from you about your opinions on decreasing the amount of chemotherapy for patients with breast cancer and how to talk to patients about participating in a trial of less chemotherapy that is usually given. Please know that there are no right or wrong answers here today. We want to understand different experiences and perspectives on this treatment approach. Please be open and candid with us. Please attempt to avoid using your name or your doctors’ names during the interview.

Before we start, I want to tell you that everything you say during your conversation with me today will remain confidential. Your responses will not be shared with your colleagues or anyone outside the research team. We may use data, without your name or other information that could identify you, in quality improvement reports and publications. Would it be OK if I record our conversation so I can best capture your responses to these questions?

Do you have any questions before we begin?

1. Would you enroll patients in a study where they received less than the usual amount of chemotherapy?
2. Why? Or Why not?
3. Are there particular patients for whom you would be reluctant? Interested?
4. What do you think would be potential barriers to participating in a trial like this one?

Probes:

1. Patients won’t like it
2. Not as interesting as trials that add treatment
3. Difficulties explaining the trial to patients
4. Fear of recurrence
5. Worry about regret if patients recur
6. Focus on doing less instead of more
7. What would have made you more comfortable engaging patients in a trial like this? Or receiving less chemotherapy as standard of care?
   1. Being told they have lower risk because of their response to treatment
   2. Avoidance of physical toxicity
   3. Logistical benefits (ability to work)
   4. Financial concerns
8. How would you explain this approach to a patient?
9. If the studies show equivalence, how comfortable will you be to apply this strategy as standard of care?
   1. Are there groups who you would not use this approach? (younger, Black)
   2. What is the amount of survival difference that you think would be acceptable?
10. Are there characteristics of the prognostic marker that you think are important?
    1. Using it regularly?
    2. Data on accuracy?
    3. Lab vs. response to treatment?
11. What additional supports or training needs would help you be more comfortable prescribing this approach?
12. How has the pandemic of COVID-19 influenced your decision making in regards to your recommendations?

Thank you very much for taking the time to participate in this study.
